# Recruitment of young women to a trial of chlamydia screening – as easy as it sounds?

**DOI:** 10.1186/1745-6215-8-41

**Published:** 2007-12-04

**Authors:** Helen Atherton, Debbie Banks, Ruth Harbit, Linzie Long, Fiona Chadd, Phillip Hay, Sally Kerry, Ian Simms, Pippa Oakeshott

**Affiliations:** 1 Division of Community Health Sciences, St George's, University of London, London, UK; 2 Department of Genitourinary Medicine, St George's Hospital, London, UK; 3 Health Protection Agency, Centre for Infections, London, UK

## Abstract

**Background:**

Recruiting to trials is complex and difficult. The Prevention of Pelvic Infection (POPI) trial aims to see if screening women for chlamydia and treating those found to be infected reduces the incidence of pelvic inflammatory disease in the following twelve months. It focuses on young, sexually active, multiethnic, mainly inner city, female students. The main aim of this paper is to describe our recruitment methods. Secondary aims in two small subgroups, are to compare characteristics of women recruited with those not recruited, and to explore participants' understanding of when their samples would be tested for chlamydia.

**Methods:**

Women students attending lectures or in common rooms at 22 universities and further education colleges were recruited by female research assistants working in pairs. Participants were asked to complete a questionnaire on sexual health and to provide self-taken vaginal swabs. In addition, during 3 recruitment sessions, a female medical student asked non-participants to complete a brief anonymous questionnaire on reasons for not taking part. Finally another female medical student contacted 40 consecutive participants within a month of recruitment and asked if they understood that their samples might not be tested for a year.

**Results:**

With enormous effort over 2 years we recruited 2526 women. A survey of 61 non-responders showed only 18 (30%) were eligible to take part (age <28, been sexually active and not been tested for chlamydia in the past 3 months). Eligible non-responders were of similar age to the 35 responders in the same recruitment sessions, but more likely to be from ethnic minority groups (67% 12/18 versus 29% 10/35 p < 0.01). Email and telephone contact with 35/40 (88%) of consecutive participants showed only two (6%) did not understand that their specimen might not be tested for chlamydia for a year. Thirty participants (85%) could name one or more possible consequences of untreated chlamydia infection.

**Conclusion:**

As in other studies, a key to attaining recruitment targets was the enthusiasm of the research team. Minority ethnic groups were probably under-represented, but understanding of participants was good.

**Trial registration:**

Clinical Trials NCT 00115388

## Background

The Prevention of Pelvic Infection (POPI) trial aims to see if screening young, sexually active female students for chlamydia and treating those found to be infected reduces the incidence of pelvic inflammatory disease (PID) in the following twelve months. It is a community based, randomised control trial (Clinical trials NCT 00115388) where samples in the intervention group are tested for chlamydia immediately, and infected women referred for treatment. Control samples are stored and analysed after 12 months. Participants are asked to fill out a consent form, complete a questionnaire on sexual health and provide self-administered vaginal swabs in the nearest toilet. A follow up questionnaire is used to assess incidence of PID in both groups a year after participation.

Recruiting participants to randomised control trials is often difficult, and around two thirds of trials fall short of their target numbers or need to extend the recruitment period (1). Recruitment has been particularly difficult in the group we wished to attract for this trial: young, multiethnic sexually active female students from 11 Further Education (FE) colleges and 11 universities in Greater London. A potential barrier was the fact that only half the samples would be tested for chlamydia immediately.

We described initial recruitment problems in the first few months of this trial and the methods developed to try and overcome them [1]. Recruitment took place over two academic years 2004–6, and much more has been learned. The main aim of this paper is to describe difficulties encountered and strategies devised to increase recruitment during the second year 2005–6 when the majority (1800) of the 2526 participants were recruited. Secondary aims in two subgroups are to compare characteristics of women recruited with those not recruited, and to explore participants' understanding of when their samples would be tested for chlamydia.

## Methods

### Recruiters

From September 2005 to October 2006 recruitment was carried out by two full time, young, recently graduated, female research assistants HA and DB. Another full time female research assistant joined the recruitment team for 6 months in January 2006. These research assistants were specifically chosen as recruiters of similar age and of the same sex as participants. Recruiters worked in teams of two or three depending on the numbers of women likely to be encountered at an institution. Female students were also trained as peer recruiters and used when more help was required, for example in lectures with more than 100 students.

### Recruitment methods

To be eligible for the trial, potential participants had to be female, aged <28, been sexually active at least once, and not been tested for chlamydia in the past 3 months. We used two recruitment methods: firstly talking to female students at the end of their lecture for five minutes to see whether they would like to take part, and secondly setting up a recruitment stall usually in a common room or reception area. When speaking to students at the end of a lecture, this was pre-arranged with the lecturer and we were often given an introduction. Where possible we focused on courses with more female students such as nursing, childcare and beauty therapy. Male students were asked to leave before a brief talk was given and information sheets handed out. After talking individually to recruiters, any female students who wished to take part did so there and then by completing a consent form and questionnaire in the lecture theatre, and providing specimens in the nearest lavatory.

When setting up a stall we liaised with the university or college to find a suitable area. We used bright posters, a pink table cloth and colourful informative fliers to help attract attention. This method usually involved us approaching groups of female students to see if they would like to take part. The two methods were used in conjunction to maximise recruitment on any particular day.

### Ethical Issues

Ethical approval for the trial was granted by Wandsworth Research Ethics Committee (Ref 03.0054). We ensured that all participants were explicitly aware that their sample might fall into the control group and not be tested for 12 months. We recommended that if they had been at risk of sexually transmitted infection they should seek testing at a health care facility. This information was in both the patient information sheet and consent form. It was also reiterated at the point of recruitment. After recruitment a copy of the patient information sheet was posted to the participant's home.

### Survey of non-responders

This was conducted as a medical student project by LL during 3 recruitment sessions in September 2006. The sessions were chosen to fit in with her academic timetable, and included both university and FE college students to give a range of potential participants. After a short explanation, LL gave a brief anonymous questionnaire to women who were not recruited. The questionnaire asked for age and ethnicity, whether students were eligible to take part and reason for non-participation.

### Survey of participants' understanding about delayed chlamydia testing

This was a student project done by another female medical student FC. In October 2006 she emailed a short questionnaire to 40 consecutive participants recruited in September 2006. The email was sent within a week of their participation. For non-responders and those without an email address she conducted the survey by telephone. The questionnaire asked:

1. Was it made clear to you that your sample may not be tested for chlamydia for a year?

2. What may happen if chlamydia infection is left untreated in women?

## Results

### Recruitment rates

**Figure 1:**
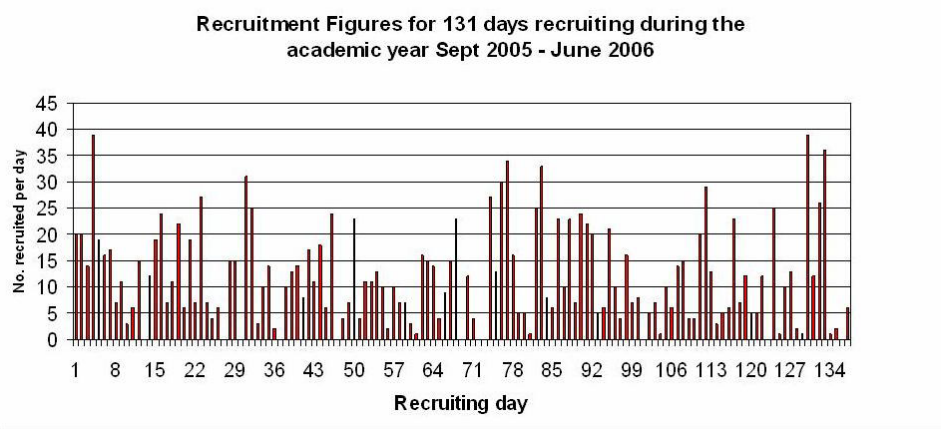
Recruitment figures for 131 days recruiting during the academic year September 2005 to June 2006.

We recruited 2526 eligible women by October 2006. Figure 1 shows that the number of women recruited per day during the second year of recruitment varied from 0–37. The days with low or zero recruitment were disappointing and bad for recruiters' morale. Reasons included cancelled lectures, the lecturer forgot we were coming, too few eligible students or too few women, or one or two negative students putting off the group.

### Characteristics of non-responders

A survey of 61 non-responders showed only 18 (30%) were eligible to take part. They were compared with 35 female students recruited at the same time. Eligible non-responders were slightly younger than responders in the same recruitment sessions 20.1 versus 21.6 years (difference 1.5 years 95% CI -0.7 to 3.7). However they were more likely than responders to be from ethnic minority groups: (67% 12/18 versus 29% 10/35 RR 2.3 (1.3 to 4.3)). Reasons given for not taking part were: feeling uncomfortable about doing a self-taken vaginal swab (6), menstruating (6), in a rush (3), no reason given (3).

### Understanding of delayed chlamydia testing

Email or telephone contact with 35/40 (88%) of consecutive participants showed that only two (6%) did not understand that their specimen might not be tested for chlamydia for a year. Thirty participants (85%) could name one or more possible consequences of untreated chlamydia infection.

## Discussion

### Principle findings

For successful recruitment we tried to be flexible and creative. However recruitment remained difficult, and our small sub study suggested that in those 3 recruitment sessions women not recruited were more likely than participants to belong to ethnic minority groups. In our other sub study understanding of the trial was good and only two of 35 participants responding said they did not understand that their vaginal samples might not be tested for chlamydia for a year.

### Strengths and weaknesses

By continually trying to improve our recruiting methods, increasing the recruitment period and number of recruiters, and extending to more institutions, we finally recruited over 2500 women. A strength of the study is that unlike most studies of sexually transmitted infections, participants were recruited outside of health care facilities. A limitation is that the substudies looking at the characteristics of non-responders and the understanding of participants only included small numbers of women and may not be representative.

As in many other trials [2], our description of what we thought worked is only subjective and could not be assessed. However three aspects deserve discussion: awareness of cultural sensitivities, user-friendly language and incentives. Approximately 40% of our participants were from ethnic minorities and attitudes to being sexually active varied. Some women did not want to admit to being sexually active; others did not want to admit that they were not. We did not want anyone to be embarrassed so we quoted our eligibility criteria together: "*In order to take part you need to have had sex at least once and not have been tested for chlamydia in the last year*". The two criteria were mutually exclusive and so the women did not have to tell us or their friends their reason for not taking part. Despite this we had to exclude 10 participants whose responses to the confidential questionnaire indicated they had never been sexually active. This highlights the possible role of peer pressure

The second aspect which we modified over the course of the study was user-friendly terminology. We talked about preventing pelvic inflammatory disease and possible infertility rather than focusing on chlamydia testing and associated stigma [3]. We also ensured that we always referred to the vaginal swab as 'a small cotton bud, like the kind you use to put on make up, and you use it just like a tampon'. Thirdly we offered small incentives in the form of lollipops and coloured pens. Increasingly participants are expecting a reward for helping with research [4] and these incentives were well received. We also appealed to participants' possible altruistic side by emphasising the important contribution participants might make to research into women's health by giving just 10 minutes of their time.

### Comparison with other studies

In their description of a condom promotion trial, Gabbay et al point out the difficulty of recruiting healthy volunteers to primary care and community based trials [5]. They noted that altruism is a powerful motivation. In a qualitative study Featherstone et al observed that most eligible patients, whatever their level of knowledge, will struggle to make sense of their participation in randomised trials [6]. Most of our sample of participants understood about the possibility of delayed chlamydia testing. Finally Ross et al conducted a systematic review of barriers to participation and suggested that the recruitment aspects of a trial should be carefully planned and piloted [7]. As in our trial, dedicated research staff are essential, and demands on patients should be kept to a minimum. Although it is not often feasible, we agree that proper evaluation is required of strategies to overcome barriers.

## Conclusion

We suggest it may be possible to use further education colleges and universities more frequently for community based research as they provide a large, varied and interesting population with which to work. Chlamydia testing using vaginal swabs is a sensitive topic, and we found it better to accept that recruitment was always going to be difficult regardless of technique, especially in a randomised trial where not every participant will be tested immediately.

Our experience may be transferable to other studies involving recruitment of young people in the community, and also to chlamydia screening in primary care. The risk of chlamydia is higher among those less likely to engage in screening [5], particularly sexually active teenagers. To be generalisable, research should make an effort to include such high risk, hard to reach groups.

## Competing interests

The author(s) declare that they have no competing interests.

## Authors' contributions

HA was involved in recruitment of the participants and the development of recruitment methods and drafted the manuscript, DB was involved in recruitment of the participants and the development of recruitment methods and was involved in drafting the manuscript, RH was involved in recruitment of the participants and the development of recruitment methods, LL carried out the survey of non responders, and was involved in drafting the manuscript, FC carried out the survey of participants understanding, and was involved in drafting the manuscript, PH participated in study design, conceived the idea of recruitment at universities and further education colleges and was involved in drafting the manuscript, SK participated in study design, provided the statistical analysis and provided substantial input in the writing of the manuscript, IS participated in the design of the study and commented on the manuscript and PO conceived the study, participated in its design and coordination and drafted the manuscript with HA. All authors read and approved the final manuscript.
